# Serological and molecular evaluation of Senecavirus A (SVA) in pigs from farrow-to-finish farms in Minas Gerais, Brazil

**DOI:** 10.1007/s42770-026-01970-4

**Published:** 2026-05-27

**Authors:** Júnio César Santos, Emelly Barbosa Calheiros, Leonardo Teófilo Toledo, Ugonna Henry Uzoka, Fernanda Simone Marks, Wagnner José Nascimento Porto, Alessandra Abel Borges, João Alberto Farinelli Pantaleão, Maria Isabel Isabel Maldonado Coelho Guedes, Carlos Eduardo Real Pereira, David Germano Goncalves Schwarz, Abelardo Silva-Júnior

**Affiliations:** 1 Department of Veterinary Medicine, Federal University of Viçosa, Viçosa, Brazil; 2 Institute of Biological and Health Sciences, Federal University of Alagoas, Maceió, Brazil; 3 Department of Preventive Veterinary Medicine, Federal University of Minas Gerais, Belo Horizonte, Brazil; 4 Department of Veterinary Medicine, Santa Catarina State University, Lages, Brazil

**Keywords:** Antibodies, Pigs, Senecavirus A, Vesicular diseases, Viral RNA

## Abstract

The emergence of Senecavirus A (SVA) as a causative agent of vesicular diseases in pigs is noteworthy, since it was identified in 2014 through outbreaks in Brazil. The SVA belongs to the *Picornaviridae* family and is a non-enveloped virus with the ability to adapt to the environment. As the virus’s stability allows it to persist on farms post-outbreak, maintaining stringent biosecurity measures is crucial for effective control. This study aims to evaluate antibody response and viral RNA shedding in swine farms. Blood and fecal samples were collected from pigs in 2022 on farms that experienced outbreaks in 2015 and 2016. The sampled pigs were from farrow-to-finish farms without clinical signs of SVA obtained from five farms in Minas Gerais state, Brazil, covering all production phases: sows, farrowing, nursery piglets, growing, and finishing pigs. Real-time PCR and serum neutralization tests were conducted. Antibodies against SVA were detected in all production phases from four of the positive farms, with sows showing the highest antibody prevalence. Viral RNA was found in fecal samples from two farms, with the farrowing phase pigs exhibiting the highest viral RNA positivity. These findings indicate the presence of SVA infection on farms with no clinical signs, suggesting that the virus may have persisted following previous outbreaks due to its environmental stability. This study provides a comprehensive profile of antibody and viral RNA presence across different production phases, which may be applicable for informing biosafety measures.

**Supplementary Information:**

The online version contains supplementary material available at 10.1007/s42770-026-01970-4.

## Introduction

Highlighting vesicular diseases in pigs is crucial due to mandatory notification requirements, particularly when foot-and-mouth disease (FMD) is suspected. Senecavirus A (SVA) infection is a significant vesicular disease and a vital differential diagnosis for FMD, requiring confirmatory laboratory tests [1]. Rapid differentiation between these diseases is essential for implementing control measures and preventing FMD introduction in disease-free areas. Furthermore, SVA outbreaks can lead to significant economic losses, as pigs from affected farms cannot be transported to slaughterhouses while clinical signs persist [2].

In 2023, the International Committee on Viral Taxonomy officially named the SVA, as the species *Senecavirus valles*, belonging to the *Picornaviridae* family [3]. SVA was initially discovered from contaminants in Per C6 cells [4]. This small virus consists of a single-stranded, positive sense RNA genome of 7.2 kb, is non-enveloped, and exhibits high environmental stability [5].

In Brazil, the presence of SVA was detected for the first time in 2014 when some brazilian states experienced outbreaks in pig farms [6]. In 2018, a new wave of the disease occurred in the states of Minas Gerais, Goiás, São Paulo, Mato Grosso, Paraná, Rio Grande do Sul, and Santa Catarina [7]. In addition to Brazil, important pig-producing countries such as Canada, China, Colombia, the USA, Thailand, and Vietnam also reported the detection of the virus [8]. In Brazil’s main swine-producing states, a retrospective study analyzed pig serum samples collected between 2007 and 2016, with the results indicated that there was no presence of SVA in Brazil before 2014 [9].

The clinical signs are identical to other vesicular diseases, with such signs including vesicular lesions and ulcerations in the oral cavity, muzzles, and coronary bands [6]. Additionally, there are reports of neonatal mortality, weight loss, culling syndrome, and diarrhea, culminating in economic losses and health obstacles for producers and slaughterhouses [10]. The disease is also related to stress and decreased immunity, which can both favor the circumstances of SVA infection [11].

Experimental vaccines have been developed in previous studies [12–14]. Nevertheless, implementing other biosecurity measures is crucial for disease prevention. Recommended actions include delineating the production unit with appropriate fencing, regularly cleaning facilities, controlling farm vehicle traffic, managing rodent and insect populations, and acquiring pigs confirmed as disease-free. Furthermore, isolating suspected animals and promptly notifying the Official Veterinary Service is mandatory within current legislation [2].

Consideration must be given to the virus’s significant environmental stability, known to be characteristic of picornaviruses [4]. The subclinical manifestation of the disease facilitates its spread among pig farms, making this a very concerning factor for allowing the virus’s persistent presence in naturally infected farms. This study aims to assess the serological status and viral shedding of SVA in pig farms that experienced outbreaks in 2015 and 2016 but exhibited no clinical disease at the time of sample collection in 2022. To achieve this, the prevalence of antibodies against SVA across different breeding phases of the pig farms was evaluated, in addition to detecting and genetically characterizing the viral RNA in farrow-to-finish farms.

## Materials and methods

### Farms and samples

The study has been approved by the Animal Ethics Committee of the Federal University of Viçosa via process number 66/2021. Blood and fecal samples from 500 pigs were collected between March and July 2022, then distributed among five farms without the occurrence of clinical disease at the time of sampling: located in the cities of Urucânia (Farm 1), Teixeiras (Farm 2), Rio Casca (Farm 3), Jequeri (Farm 4), and Ponte Nova (Farm 5) – all situated in Minas Gerais State, Brazil, where episodes of SVA occurred between 2015 and 2016 and were diagnosed by the Official Federal Veterinary service. In each of these farms, samples were collected from 100 animals and then subdivided into 20 animals from each phase: sows, farrowing piglets (0–21 days of age), nursery piglets (22–60 days of age), growing pigs (61–90 days of age), and finishing pigs (91–130 days of age).

### SVA serology

The SVA strain (SVALPVA 4) was given by the School of Veterinary, from the Federal University of Viçosa, Minas Gerais, and was used as the standard virus for serum neutralization assays. Cells of the NCI-H1299 lineage (ATCC^®^ CRL-5803TM) were multiplied and maintained at 37 °C in a 5% CO_2_ atmosphere using modified RPMI-1640 medium containing 2 mM L-glutamine, 10 mM HEPES, 1 mM sodium pyruvate, 4,500 mg/L glucose, 1,500 mg/L sodium bicarbonate, and 10% fetal bovine serum.

Serum neutralization assays were performed as described by House & Baker [15], adding 100 TCID_50_/50 µL of SVALPVA 4 strain to the serum dilutions (1:4 to 1:32768) of each sample serum. After sera-virus incubation for 1 h at 37 °C in a 5% CO_2_ atmosphere, 50 µL of NCI-H1299 cell suspension was added at a concentration of 3 × 10^4^ cells. Results analysis was performed after 72 h of incubation by monitoring the cytopathic effect. The neutralizing activity of the anti-SVA antibody was expressed as the geometric mean of the observed values. Positive and negative reference samples are used as controls.

### Viral RNA detection

Viral RNA was extracted from feces using the BioGene Viral DNA/RNA Extraction Kit (Bioclin, Brazil), according to the manufacturer’s recommendations. To obtain cDNA, reverse transcription was performed. The GoScriptTM Reverse Transcription System kit (Promega, USA) was also utilized according to the manufacturer’s instructions.

Real-time PCR occurred using genetic material extracted from collected feces and subjected to reverse transcriptase. Primers and methodology were adapted for use [16]. The RT-qPCR assays were based on GoTaq^®^ qPCR Master Mix (Promega, USA). Amplifications were performed using the Rotor-Gene 5-Plex HRM real-time PCR detection system (Qiagen, USA), Software Version 2.3.1.

### Nucleotide sequencing and bioinformatic analyses

Undertaking the Sanger method, nested-PCR products obtained from five positive samples were subjected to nucleotide sequencing in quadruplicate using the BigDyeTM Terminator Kit (Thermo Fischer Scientific, USA) and the ABIPrism 3500 Genetic Analyzer (Applied Biosystems, USA). From the RNA, the GoScriptTM Reverse Transcription System kit (Promega, USA) enabled cDNA synthesis. Primers and reaction conditions were used [17], allowing the amplification of a region referring to the genetic fragment of the VP1 structural protein, represented by 316 base pairs.

Sequence editing and *denovo* assembly were performed using the computer program Geneious v2023.1 [18]; an additional 47 sequences available in GenBank were downloaded. Sequences were aligned using MAFFT v.7 [19] with default parameters. The Maximum Likelihood (ML) approach was performed with the 2-parameter Kimura model [20] in MEGA X [21].

### Statistical analysis

The data were analyzed using absolute frequencies and arithmetic means. To evaluate the association between categorical dependent variables (seropositivity or PCR-positive) and independent variables (different categories such as farrowing, nursery, growing, and finishing) either the Person chi-square test (serology) or the Fisher’s exact test was used, depending on the test requirement. Statistical analyses of quantitative variables were performed using the one-way ANOVA test and, to assess the homoscedasticity of the data, the Bartlett test was performed. To evaluate the relationships between the means of the total categories, Tukey’s multiple comparison test was performed. Furthermore, to evaluate associations between the risk of two categories (bivariate) of production passing infection – sows, farrowing, nursery, growth, and finishing – a dichotomous comparative evaluation was carried out with the aim of obtaining an odds ratio (OR) when performing the chi-square or Fisher’s exact test. All statistical analyses and graphs were performed using the GraphPad Prism^®^ version 8.0.2 program, considering a 95% confidence interval and *P* ≤ 0.05.

## Results

### Serology profile against SVA

**Fig. 1:**
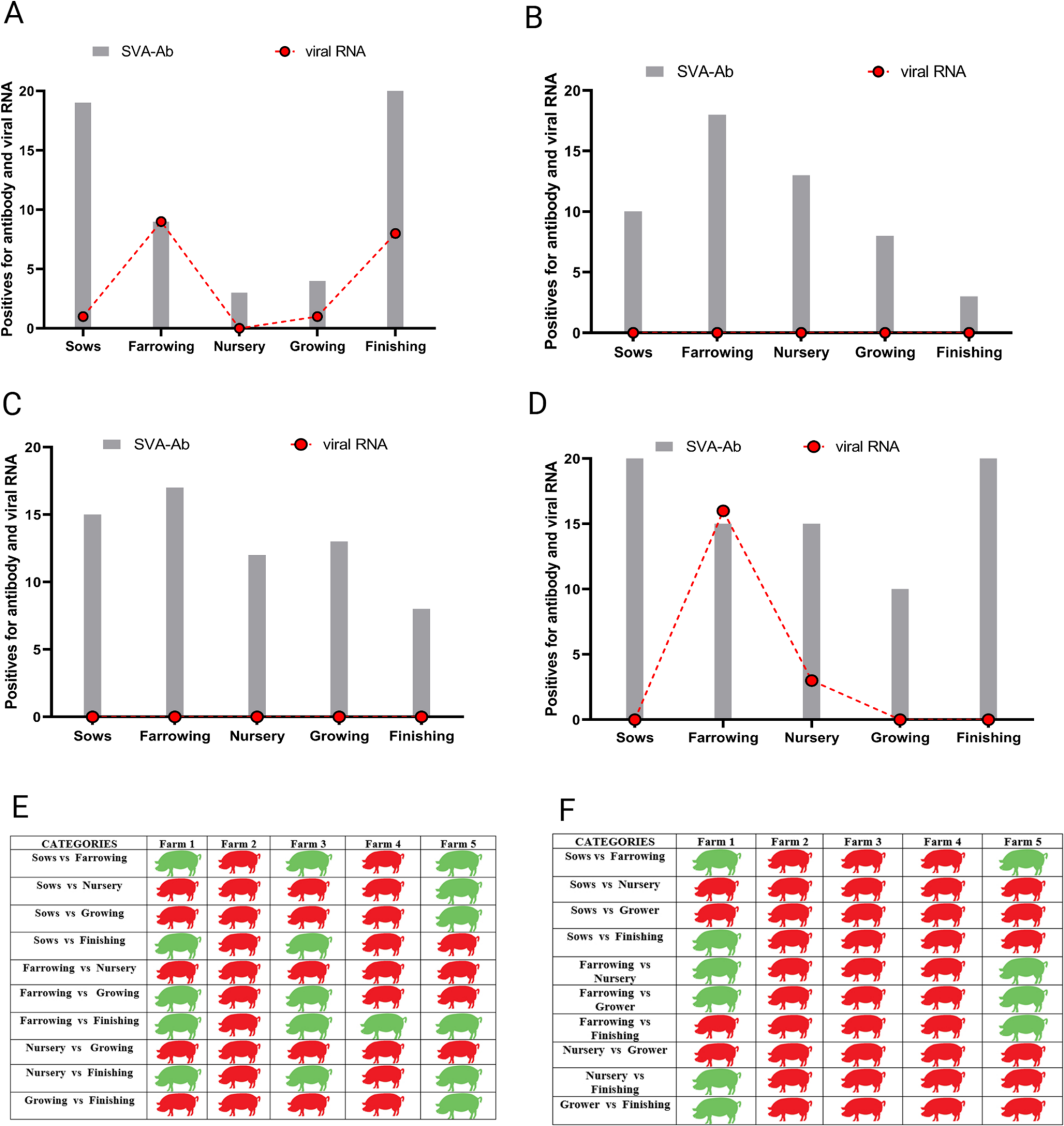
Serological and viral RNA detection by categories of sows, farrowing crates, piglets nursery, growing pigs, and finishing pigs from farms 1, 3, 4, and 5. The vertical axis represents the number of seropositive animals and the number of animals positive for viral RNA. **A**: Farm 1;**B**: Farm 3; **C**: Farm 4; **D**: Farm 5; **E** and **F**: represent statistical differences considering each category, together with farms for serology and qPCR results, respectively. The color green represents statistical difference. The color red represents no statistical difference

Four farms showed antibodies against SVA. Farm 2 (Teixeiras), however, did not show antibody detection in the serum neutralization assay. In Fig. 1, observing the serological profile is possible. This enables a better understanding of the qualitative serological positivity found on each farm, including within their respective production phases. No standard serological response exists among farms; hence, it must be considered that farms have different management systems and protocols influencing the potential virus distribution throughout the various breeding phases.

Farm 1 had 55% total serological positivity (55/100), comprising 19 sows (19/20, 95%), 9 farrowing piglets (9/20, 45%), 3 nursery piglets (3/20, 15%), 4 growing animals (4/20, 20%), and 20 finishing pigs (20/20, 100%). Farm 3 demonstrated 52% (52/100) of total positivity, with 10 sows (10/20, 50%), 18 farrowing piglets (18/20, 90%), 13 nursery piglets (13/10, 65%), 8 growth animals (8/20, 40%), and 3 finishing animals (2/20, 15%). On Farm 4, 65% of positive animals were obtained (65/100), distributed in 15 sows (15/20, 75%), 17 farrowing piglets (17/20, 85%), 12 nursery animals (12/20, 60%), 13 growing animals (13/20, 65%), and 8 finishing animals (8/20, 40%). Farm 5 presented a percentage of 80% total positivity (80/100) and, of these animals, 20 were sows (20/20, 100%), 15 farrowing piglets (15/20, 75%), 15 nursery piglets (15/20, 75%), 10 growing pigs (10/20, 50%), and 20 finishing animals (20/20, 100%).

In the comparison made between the farms’ production phases – illustrated in Fig. 1E – it is possible to observe images in green showing a statistical difference and images in red showing no such difference. Heterogeneity clearly exists between farms. Nevertheless, between phases of nursery vs. growing and farrowing vs. nursery, we can detect no difference in any positive farm regarding the number of positive pigs for antibodies anti-SVA. (On Farm 2, this analysis was irrelevant because it was a negative farm.)

**Fig. 2:**
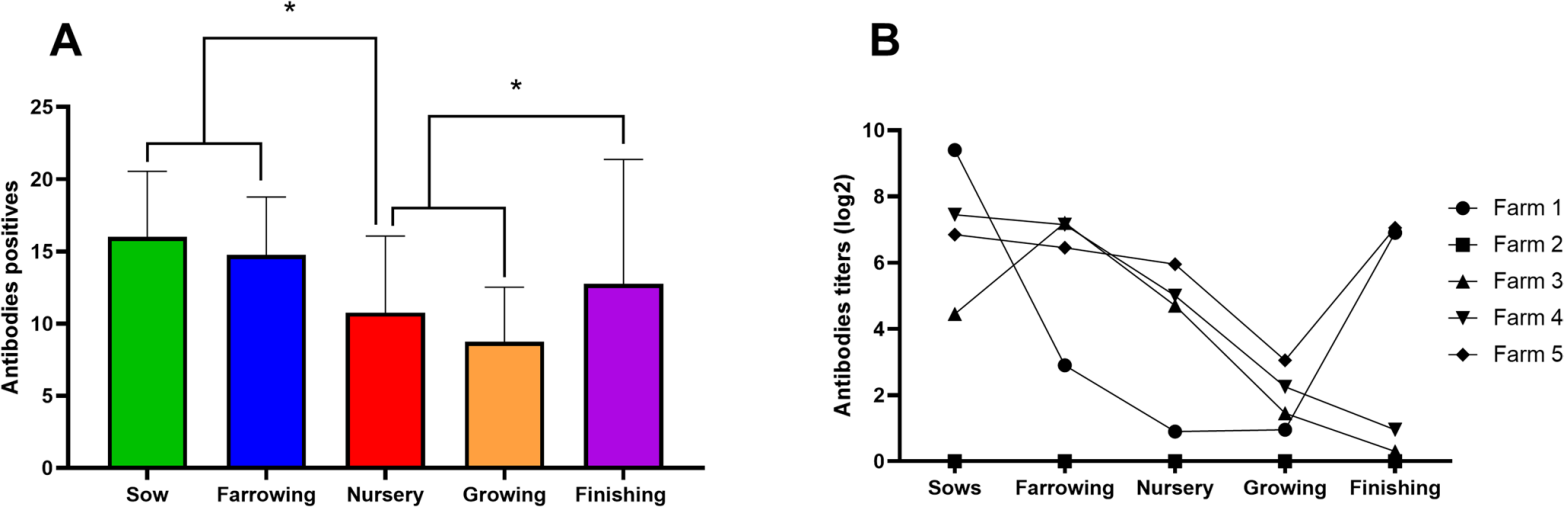
Profile of antibodies response by categories (sows, farrowing crate, nursery piglets, growing pigs, and finishing pigs). **A**: Average number of positive pigs considering all positive farms by categories. **B**: Average of antibodies titers in log2 in each farm by categories

When considering the analysis and Fig. 2 A, which shows the positive and negative antibody data from all positive farms (1, 3, 4, and 5), it is possible to see that sows and farrowing present a greater number of individuals showing the presence of antibodies with significant statistical differences when compared to pigs from nursery and growing phases (*p* < 0.05). Additionally, the pigs from the finished phase were more positive for anti-SVA antibodies than those from the nursery and growing phases (*p* < 0.05).

In the quantitative analysis of antibody titers determined by the serum neutralization method and stratified by farm, significant results variability was observed (Fig. 2B). On Farm 1, antibody titers ranged from 4 to 16,384, with the highest concentrations found in sows and finishing pigs. Farm 3 exhibited titers from 4 to 8,192, with the farrowing piglets showing the highest levels. On Farm 4, elevated titers were noted in both sows and farrowing piglets. Similarly, Farm 5 showed the highest antibody levels in sows and finishing pigs, with similar titers observed across the sow, nursery, and finishing phases (Fig. 2B). Table S1 shows the number of positive animals via the serological assay as well as the titers found in the different categories.

## Shedding of RNA SVA from feces samples

Positivity for viral RNA was obtained through real-time PCR on farms 1 and 5. In contrast, farms 2, 3, and 4 were considered negative for the detection of viral RNA (Fig. 1 A and D). On Farm 1, positivity was detected in a total of 19% of the animals (19/100), including 1 sow (1/20), 9 farrowing piglets (9/20), 1 pig in the growth phase (1/20), and 8 finishing-phase pigs (8/20). On Farm 5, positivity was also found in the feces of 19% of the animals (19/100), including 16 farrowing piglets (16/20), and 3 piglets from the nursery phase (3/20). On the other farms studied, viral RNA was not detected from the fecal samples collected (Fig. 1A–D). Table S2 shows the number of positive animals by real-time PCR across the different phases on farms 1 and 5. 

Concerning farrowing, there were some statistical differences in this category, for which farms 1 and 5 are the more affected (*p* < 0.05). In Farm 5, however, the qPCR technique also showed greater detection of viral RNA in the finishing phase (*p* < 0.05) (Figs. 1 A, D, and F).

### Genetic characterization

**Fig. 3:**
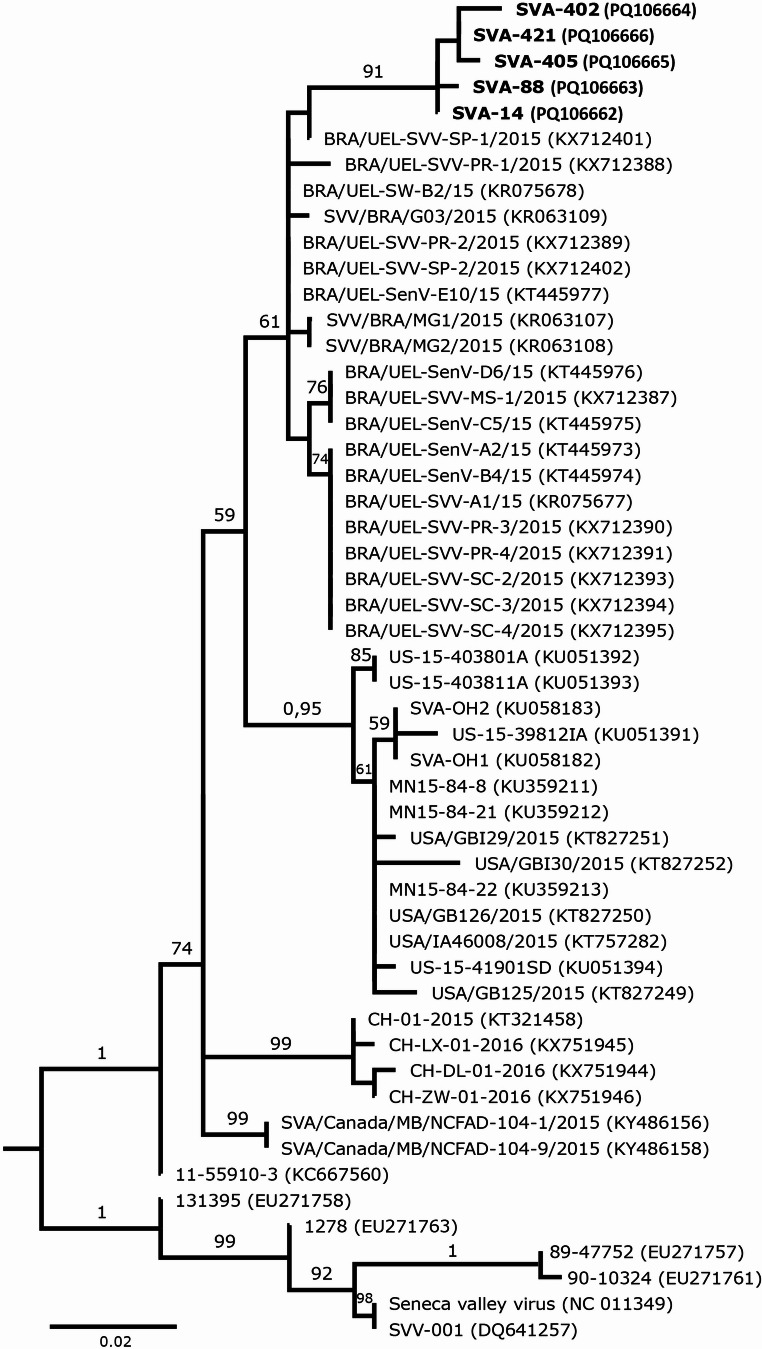
Phylogenetic tree based on the Maximum Likelihood method constructed with the two-parameter Kimura model based on the partial nt sequence of the VP1 genomic region of the Senecavirus A strains obtained in this study (sequences in bold) and others available in GenBank. GenBank accession numbers are given in parentheses. Numbers in branches refer to significance values ​​from 1,000 bootstrap replicates. Values​​≤ 50% are not shown. Genbank access: PQ106662 (SVA-14), PQ106663 (SV-88), PQ106664 (SV-402), PQ106665 (SV-405), and PQ106666 (SV-401)

Five samples positive for viral RNA were selected for sequencing: two from Farm 1 and three from Farm 5. A 316-bp fragment referring to the partial sequence of the VP1 protein was sequenced. Through sequencing analysis of the amplicons obtained, confirming that this fragment referred to Senecavirus A was possible. Strains SVA-14 and SVA-88 originate from farrowing crates and sows of Farm 1, respectively. The strains SVA-402 and SVA-405 are from the piglet crates of Farm 5, while SVA-421 came from a piglet nursery of Farm 5. The SVA-14 and SVA-421 strains show a 99.7% identity with the Brazilian sample MZ032152, while the SVA-88, SVA-405, and SVA-402 show identities of 99.5%, 99.3%, and 99.2% with sample MZ032152, respectively. The phylogenetic tree indicates that the region of the viral genome is genetically similar to Brazilian strains, with the strains analyzed in this study being more closely related to each other than to other sequences originating from Brazil (Fig. 3).

## Discussion

Since the virus can affect the health and well-being of animals, infections caused by SVA are important for swine farming, resulting in considerable zootechnical and economic losses. SVA should be understood as a vesicular disease, requiring essential procedures in the production and health chain of animal-origin products. Managing these various challenges represents a great difficulty brought about by a small, non-enveloped virus that is difficult to control, with no age predilection, an environmental resistance profile, and the obvious potential for perpetuation on farms [22].

This study provides important insights regarding farms naturally exposed to SVA, highlighting the challenges posed by unvaccinated herds that did not exhibit clinical symptoms of SVA infection. Also, the virus can persist subclinical within the herd, and the animals can experience viral shedding and viremia [23]. This suggests that the virus persisted within the herd until the sample collection dates in March and July 2022. It should be noted that the clinical record of the disease being present in these herds was recorded by the health authorities as 2015 and 2016. At that time, the region studied was experiencing a major SVA outbreak [7].

According to real-time PCR analysis, the positivity detected in swine from farms 1 and 5 is explained by the excretion of viral RNA in the collected feces, which aligns with the finding that described viral excretion through nasal, fecal, and oral routes [24]. The presence of the virus in facilities, the environment, flies, and other animal species – highly efficient transmission vehicles – poses significant challenges to controlling and eradication of the virus, which is already endemic in the country [25].

Regarding the phases in which serological positivity for viral infection was observed, detecting a marked presence in sows is possible. These are animals housed for long periods, particularly in farms 1 and 5. This swine category faces highly challenges and exposure to pathogens on the farm. It is also important to consider the high turnover required in the production phases. Although there are reports of diseases caused by SVA in pigs of different stages [26], the study clearly finds that the sows were at the highest risk.

As a transient infection [27], it is plausible that not all animals exhibit signs of viral excretion, as the infection may have ceased before the collection of biological samples for this study. Hence, the positivity found in the tests is justified by the fact that the animals had prior exposure to the virus, leading to an immunological response at different intensities.

Notably, Farm 2 did not present positive animals. In a study, was observed an immunological response to SVA caused by the production of neutralizing antibodies from the 5 d.p.i. to the 38 d.p.i [24]. Interestingly, other study reported that, after 180 days of infection, was observed a decline in antibody levels of some animals [28]. In this sense, it is reasonable to infer that there was no SVA exposure at the time of the study for at least 180 days before sample collection on the farms, considering that this farm was already repopulated with animals different from those challenged by the disease. Furthermore, the importance of quality biosecurity management to eradicate the disease is highlighted, even though the virus can be endemic [29, 30]. Still focusing on Farm 2, however, it should be highlighted that, according to our assessment, this farm demonstrates excellent health management. This raises the question of whether such practices may have contributed to the lack of positive animals among the sampled population.

In the case of serologically positive animals during the farrowing phase, the detection of antibodies originating from passive immunity through colostrum is highly likely, although this does not rule out the occurrence of natural infection or immunological response in the animals studied [11]. In the nursery piglets, a critical point to be analyzed is the intensity of stress to which they are exposed; a sudden change in its environment, combined with the separation from the mother and interaction with other individuals in the stalls may be crucial. It is known that stress is a predisposing factor for various infections, and SVA is no exception. There is the possibility of immunosuppression and weakened humoral responses, although high titers were still detected in this breeding phase, especially on farms 3 and 4 [31].

A decline in the number of serology-positive animals during the growing animal phase can be justified by the decay of probable maternal antibodies [1]. For animals during the finishing phase, their age is an important factor to consider, as their farm trajectory allows a likelihood of exposure to viral infection, with the subsequent production of neutralizing antibodies. In this sense, it is noticed that antibody levels between the growth and finishing phases increase in farms 1 and 4.

It is important to emphasize that the individuality of each animal and the environment to which it is exposed closely linked to its serological profile on a given farm [11]. This may explain the variation in the serological profiles observed across farms found in this research.

Sequencing analysis reveals that the viral RNA detected in the experimental animals is consistent with the reality of the epidemiological region. It aligns with the Brazilian strains, which have certainly been circulating since their detection in the country, challenging several pig farms and often leading to vesicular diseases. However, the strains identified in our study shows a position in a different clade.

Our study confirms the presence of SVA infection on farms with previous outbreaks, even in the absence of clinical signs. This suggests that the virus may be silently circulating within these farms. Evidence for this includes the detection of viral RNA in the animals and the antibody response observed, except on Farm 2. Our analysis reveals higher antibody levels in sows compared to other categories. This antibody profile across different production stages provides valuables insights into the virus’s circulation in pigs and can contribute to the development of biosecurity measures. We recommend ongoing monitoring farms with a history of SVA, even in the absence of clinical signs, to prevent the virus from spreading to unaffected areas.

## Supplementary Information

Below is the link to the electronic supplementary material.

Supplementary Material 1 (DOCX 22.5 KB)

Supplementary Material 2 (DOCX 14.0 KB)

## Data Availability

The sequences reported in this study have been deposited in the GenBank database. The specific accession numbers for all generated sequences are provided in Fig. 3.
