# Supplementary material for: Serological and molecular evaluation of Senecavirus A (SVA) in pigs from farrow-to-finish farms in Minas Gerais, Brazil
Source: Braz J Microbiol. 2026 May 27;57(1):158. doi: 10.1007/s42770-026-01970-4 (PMC13216374; doi:10.1007/s42770-026-01970-4)
Supplement: Supplementary file 1 — Supplementary Material 1 (DOCX 22.5 KB) [file 42770_2026_1970_MOESM1_ESM.docx]

|  | **Farm 1** | | | | | **Farm 2** | | | | | **Farm 3** | | | | | **Farm 4** | | | | | **Farm 5** | | | | |  |
| --- | --- | --- | --- | --- | --- | --- | --- | --- | --- | --- | --- | --- | --- | --- | --- | --- | --- | --- | --- | --- | --- | --- | --- | --- | --- | --- |
| **Ab titers** | A | B | C | D | E | A | B | C | D | E | A | B | C | D | E | A | B | C | D | E | A | B | C | D | E | |
| <8 | 1 | 11 | 17 | 16 | 0 | 20 | 20 | 20 | 20 | 20 | 10 | 2 | 7 | 12 | 17 | 5 | 3 | 8 | 7 | 12 | 0 | 5 | 5 | 10 | 0 | |
| 8 | 0 | 1 | 0 | 0 | 0 | 0 | 0 | 0 | 0 | 0 | 1 | 3 | 1 | 2 | 3 | 1 | 0 | 0 | 7 | 6 | 0 | 0 | 0 | 1 | 1 | |
| 16 | 1 | 0 | 0 | 2 | 1 | 0 | 0 | 0 | 0 | 0 | 0 | 0 | 3 | 2 | 0 | 0 | 1 | 2 | 0 | 1 | 3 | 0 | 2 | 1 | 0 | |
| 32 | 0 | 0 | 0 | 0 | 0 | 0 | 0 | 0 | 0 | 0 | 0 | 0 | 1 | 1 | 0 | 0 | 2 | 0 | 1 | 1 | 1 | 0 | 1 | 2 | 1 | |
| 64 | 0 | 1 | 2 | 1 | 1 | 0 | 0 | 0 | 0 | 0 | 1 | 0 | 0 | 3 | 0 | 0 | 1 | 0 | 3 | 0 | 1 | 1 | 0 | 0 | 3 | |
| 128 | 0 | 4 | 0 | 0 | 3 | 0 | 0 | 0 | 0 | 0 | 0 | 2 | 0 | 0 | 0 | 0 | 2 | 0 | 2 | 0 | 4 | 2 | 2 | 1 | 1 | |
| 256 | 1 | 1 | 0 | 0 | 8 | 0 | 0 | 0 | 0 | 0 | 0 | 3 | 1 | 0 | 0 | 0 | 0 | 2 | 0 | 0 | 4 | 2 | 1 | 2 | 6 | |
| 512 | 3 | 1 | 0 | 1 | 7 | 0 | 0 | 0 | 0 | 0 | 1 | 2 | 1 | 0 | 0 | 1 | 0 | 1 | 0 | 0 | 0 | 2 | 0 | 1 | 3 | |
| 1024 | 3 | 0 | 1 | 0 | 0 | 0 | 0 | 0 | 0 | 0 | 2 | 1 | 1 | 0 | 0 | 3 | 2 | 2 | 0 | 0 | 5 | 3 | 4 | 1 | 3 | |
| 2048 | 4 | 0 | 0 | 0 | 0 | 0 | 0 | 0 | 0 | 0 | 3 | 0 | 1 | 0 | 0 | 2 | 4 | 3 | 0 | 0 | 0 | 2 | 2 | 0 | 1 | |
| 4096 | 2 | 0 | 0 | 0 | 0 | 0 | 0 | 0 | 0 | 0 | 0 | 4 | 1 | 0 | 0 | 5 | 4 | 1 | 0 | 0 | 2 | 2 | 2 | 1 | 1 | |
| 48192 | 2 | 1 | 0 | 0 | 0 | 0 | 0 | 0 | 0 | 0 | 0 | 3 | 0 | 0 | 0 | 2 | 0 | 0 | 0 | 0 | 0 | 0 | 1 | 0 | 0 | |
| 16384 | 1 | 0 | 0 | 0 | 0 | 0 | 0 | 0 | 0 | 0 | 2 | 0 | 3 | 0 | 0 | 1 | 1 | 1 | 0 | 0 | 0 | 1 | 0 | 0 | 0 | |
| 32768 | 2 | 0 | 0 | 0 | 0 | 0 | 0 | 0 | 0 | 0 | 0 | 0 | 0 | 0 | 0 | 0 | 0 | 0 | 0 | 0 | 0 | 0 | 0 | 0 | 0 | |
| 65536 | 0 | 0 | 0 | 0 | 0 | 0 | 0 | 0 | 0 | 0 | 0 | 0 | 0 | 0 | 0 | 0 | 0 | 0 | 0 | 0 | 0 | 0 | 0 | 0 | 0 | |
|  |  |  |  |  |  |  |  |  |  |  |  |  |  |  |  |  |  |  |  |  |  |  |  |  |  |  |

**Table S1:** Number of positive pigs based on serum neutralization assay by titers in each category. A: represent sows; B: represent farrowing piglets; C: represent nursery piglets; D: represent growing pigs, and E: represent finished pigs.
