# Supplementary material for: Serological and molecular evaluation of Senecavirus A (SVA) in pigs from farrow-to-finish farms in Minas Gerais, Brazil
Source: Braz J Microbiol. 2026 May 27;57(1):158. doi: 10.1007/s42770-026-01970-4 (PMC13216374; doi:10.1007/s42770-026-01970-4)
Supplement: Supplementary file 2 — Supplementary Material 2 (DOCX 14.0 KB) [file 42770_2026_1970_MOESM2_ESM.docx]

| Production phase | Farm 1  Prevalence | Farm 5  Prevalence | Farm 1  (%) | Farm 5  (%) |
| --- | --- | --- | --- | --- |
| Nursing piglets | 9/20 | 16/20 | 45 | 80 |
| Nursering piglets | 0/20 | 3/20 | 0 | 15 |
| Growing pigs | 1/20 | 0/20 | 5 | 0 |
| Finishing pigs | 8/20 | 0/20 | 40 | 0 |
| Sow | 1/20 | 0/20 | 5 | 0 |
| Total | 19/100 | 19/100 | 19 | 19 |

**Table S2:** Prevalence of real time PCR-positive pigs for Senecavirus A and percentage (%) by production phase in farms 1 and 5. Nursing piglets were defined as animals aged 0–21 days, nursery piglets as 22–60 days, growing pigs as 61–90 days, and finishing pigs as 91–130 days. Sows were defined as adult females in the reproductive phase. Ct value threshold< 33,44 [16].
